# Modern approaches to radiotherapy in primary cutaneous lymphomas: insights and recommendations from the DEGRO dermato-oncology working group

**DOI:** 10.1007/s00066-025-02453-5

**Published:** 2025-09-09

**Authors:** Khaled Elsayad, Dora Correia, Ulrike Theiß, Andrea Baehr, Angela Besserer, Oliver Micke, Burkhard Greve, Cora Waldstein, Stefanie Corradini, Daniel Habermehl, Laila König, Kathrin Hering, Sebastian Adeberg, Hans Theodor Eich

**Affiliations:** 1 Department of Radiation Oncology, Philipps-University Marburg, Marburg, Germany; 2 Department of Radiation Oncology, Cantonal Hospital Aarau, Aarau, Aargau Switzerland; 3 Department of Radiotherapy and Radiation Oncology, University Medical Center Hamburg-Eppendorf, Hamburg, Germany; 4 Department of Radiation Oncology, Charité-Universitätsmedizin Berlin, Berlin, Germany; 5 Department of Radiation Oncology, Ernst von Bergmann Hospital Potsdam, Potsdam, Germany; 6 Department of Radiation Oncology, Franziskus Hospital Bielefeld, Bielefeld, Germany; 7 Department of Radiation Oncology, University Hospital of Münster, Münster, Germany; 8 Department of Radiation Oncology, Comprehensive Cancer Center, Medical University Vienna, Vienna, Austria; 9 Department of Radiation Oncology, LMU University of Munich, 81388 Munich, Germany; 10 Department of Radiation Oncology, Justus-Liebig-University, UKGM Giessen, Giessen, Germany; 11 Department of Radiation Oncology, Heidelberg University Hospital, Heidelberg, Germany; 12 Department of Radiation Oncology, University of Leipzig Medical Center, Leipzig, Germany; 13 Department of Radiation Oncology, UKGM Marburg, Marburg, Germany; 14 Marburg Ion-Beam Therapy Center (MIT), Department of Radiation Oncology, UKGM Marburg, Marburg, Germany; 15 University Cancer Center (UCT) Frankfurt-Marburg, Frankfurt, Marburg, Germany; 16 Department of Radiation Oncology, Inselspital, Bern University Hospital, University of Bern, Bern, Switzerland

**Keywords:** S-MISR-Register, Overtreatment, Quality of life, Shared decision-making, IMRT

## Abstract

The growing use of reduced-dose radiotherapy in patients with primary cutaneous lymphoma is a promising development. Nevertheless, the absence of controlled clinical trials to ascertain standardized doses for each specific type constitutes a significant impediment to the advancement of this field. This expert opinion strongly advocates for advancements in radiation oncology practice that address the unique complexities of primary cutaneous lymphoma. By refining our methodologies, we can optimize patient care and outcomes in this dynamic field.

## Introduction

Primary cutaneous lymphomas comprise a diverse group of non-Hodgkin lymphomas that are both increasingly common and multifaceted [1]. The preponderance of these cells, constituting over two thirds of the total population, originate from T lymphocytes, while the residual fraction is derived from B lymphocytes. Accurate identification of the specific type of lesion involves various methodological approaches, including histological and molecular analyses [2]. Primary cutaneous lymphomas primarily affect middle-aged and older adults, emphasizing the need for awareness and early detection [3]. Patients frequently encounter cutaneous manifestations that have been demonstrated to diminish their health-related quality of life [4–9]. The treatment strategy is individually tailored based on the stage of the disease, thus ensuring optimal patient care. In this expert opinion, we highlight the critical role of modern RT fractionation schemes and provide a clear radiation algorithm, demonstrating its significant potential to improve outcomes for several forms of the disease [10, 11].

Low-dose radiotherapy (RT) is an exceptionally effective treatment for various entities, known for its outstanding tolerability in cutaneous lymphoma [12]. However, use of conventional doses > 24 Gy still seems to be common practice among a significant number of radiation oncologists [13]. Low-dose radiation therapy not only exerts a direct cytotoxic effect but also enhances its antitumor efficacy by activating CD4+ and CD8+ T cells in the lymph nodes [14]. When combined with immunotherapy, this approach can trigger a powerful systemic response. Notably, antitumor immunotherapy showed increased effectivity when preceded by low-dose RT, which opens up promising avenues in the battle against cancer [15, 16].

Besides promising clinical advantages, modern RT doses for cutaneous lymphomas offer benefits from both patient and healthcare perspectives. It is not only more convenient but also requires fewer hospital visits, thereby effectively reducing time demands and costs [17]. The rising importance of discussion with patients undergoing RT emphasizes the pressing necessity for patient empowerment and shared decision-making.

## Current treatment modalities for cutaneous lymphoma

The European Organization for Research and Treatment of Cancer (EORTC) recommendations provide guidance on selecting RT fractionation schemes based on the type of lymphoma [12]. In this section, we give updated recommendations regarding RT dose and fractionation for the most common types of cutaneous lymphoma.

### Primary cutaneous T-cell lymphomas

**Fig. 1:**
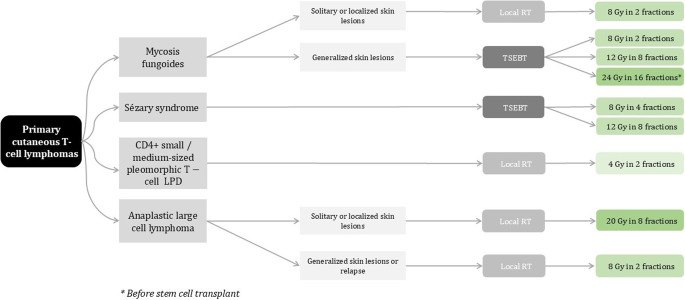
Radiotherapy dose recommendations for primary cutaneous T‑cell lymphomas (adapted from European Organization for Research and Treatment of Cancer recommendations). *LPD* Lymphoproliferative disorder; *RT* Radiotherapy; *TSEB* Total skin electron beam therapy

The radiation algorithm for cutaneous T-cell lymphomas is depicted in figure 1.

#### Mycosis fungoides

Local RT is an effective treatment for plaques and tumors related to mycosis fungoides (MF). This modality can be used as a standalone treatment for localized disease or as an adjuvant therapy for lesions that do not adequately respond to other therapies. Additionally, RT can be a significant palliative measure for patients with advanced or symptomatic lesions [12, 13]. The dose prescription is typically 8 Gy in two fractions [18, 19]. A retrospective analysis shows that a single-fraction low-dose radiotherapy of 6 Gy is equally effective as a two-fraction regimen of 2 x 4 Gy in achieving a complete response for localized MF [20]. For patients with > 10% body surface area (BSA) involvement, low-dose total-skin electron beam therapy (TSEBT) with 8–12 Gy over 2 weeks is recommended [21–26]. If the response to low-dose radiation is insufficient after 3 months, a second radiation course can be administered. Subsequent or maintenance systemic treatments after TSEBT may improve outcomes [12, 27–29].

#### Sézary syndrome

Low-dose TSEBT with 8–12 Gy over 2 weeks can improve skin symptoms and quality of life within 4–8 weeks of starting RT [8, 30]. It effectively reduces cutaneous tumor burden and lymphadenopathy [31]. In refractory cases of Sézary syndrome (SS), RT can alter the tumor microenvironment by increasing targeted receptor expression. Moreover, TSEBT is also useful for patients who require systemic treatment-free intervals due to bad general condition or side effects of other treatment modalities [12].

#### Primary cutaneous anaplastic large cell lymphoma

For patients with primary cutaneous anaplastic large cell lymphoma (PCALCL) with either solitary or multiple localized lesions, local radiotherapy at a dose of 20 Gy in 8 fractions has proven to be highly effective, leading to a low rate of relapses [32, 33]. In cases where patients have multiple lesions, administering RT as 8 Gy in two fractions can quickly relieve skin manifestations and reduce the frequency of hospital visits [33].

#### Primary cutaneous CD4+ small/medium T-cell lymphoproliferative disorder

Solitary lesions can be effectively addressed with low-dose radiotherapy, delivering 4 Gy in two fractions. Remarkably, this approach shows a complete remission rate (CRR) beginning from 92% in the existing literature, demonstrating its compelling efficacy [34–36].

### Primary cutaneous B-cell lymphomas

Primary cutaneous marginal zone lymphoma/lymphoproliferative disorder and follicle center lymphoma stand out as the most prevalent forms of indolent lymphomas, each offering a remarkably favorable prognosis [36]. Doses as low as 4 Gy in two fractions elicit CRR in over 90% of cases, as has been shown in several retrospective series. Higher doses might reach even higher CR rates and the dosage might be increased after a non-satisfying result from low-dose RT. This flexibility in dosage not only enhances treatment effectiveness but also contributes to the hope of a positive outcome for those affected [9, 12, 37, 38].

**Fig. 2:**
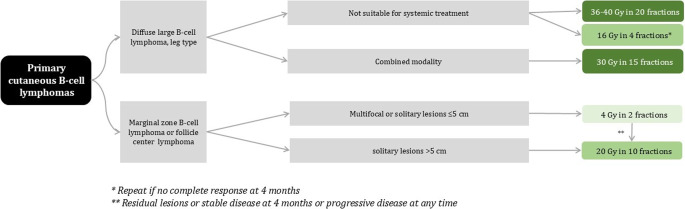
Radiotherapy dose recommendations for primary cutaneous B‑cell lymphomas (adapted from European Organization for Research and Treatment of Cancer recommendations)

Primary cutaneous diffuse large B‑cell lymphoma leg type (PCDLBCL-LT) is a rare condition that often carries a challenging prognosis. The treatment for PCDLBCL-LT artfully combines rituximab-based therapies with age-appropriate systemic therapies, showcasing a commitment to personalized care [12]. For localized cases, a consolidative radiation therapy dose of 30 Gy in 2‑Gy daily fractionated involved-site radiotherapy may be employed to maximize the therapeutic impact. For patients unable to receive systemic therapy, higher doses of conventional radiotherapy or hypofractionated radiation regimens may be considered as an option. The radiation algorithm for cutaneous B-cell lymphomas is depicted in figure 2. Recently, a collaborative analysis by the German Society of Radiation Oncology (DEGRO) and the Working Group on Dermatological Oncology (ADO) revealed that reduced-dose radiotherapy (30–36 Gy) was effective for local control in a large retrospective cohort [39]. A therapy de-escalation strategy fosters may be a reasonable option when facing challenging situations among this elderly group of patients [12, 40, 41]. Localized lesions can often be effectively treated with local radiation using electrons. However, for certain body surfaces, photon fields from intensity-modulated radiotherapy may be necessary to achieve a homogeneous dose distribution [42].

## Conclusion

Modern-dose local radiation therapy is an effective treatment for various cutaneous lymphomas, offering quick relief from skin symptoms with minimal toxicities and rare local relapses. Low-dose TSEBT also demonstrates satisfactory response rates, rapid symptom relief, and improved health-related quality of life for patients with mycosis fungoides and Sézary syndrome.
